# A Review on Current Development of Membranes for Oil Removal from Wastewaters

**DOI:** 10.3390/membranes10040065

**Published:** 2020-04-07

**Authors:** Brian Bolto, Jianhua Zhang, Xing Wu, Zongli Xie

**Affiliations:** 1 CSIRO Manufacturing, Private bag 10, Clayton South, VIC 3169, Australia; brianbolto@optusnet.com.au (B.B.); xing.wu@csiro.au (X.W.); 2 Institute for Sustainable Industries & Liveable Cities, Victoria University, Melbourne, VIC 8001, Australia; jianhua.zhang@vu.edu.au

**Keywords:** membranes, oil removal, wastewater

## Abstract

The current situation with the problems associated with the removal of oil from wastewaters by membranes is being explored. Many types of membranes have been investigated—organic polymers, inorganic or ceramic species and hybrids of the two. Polymeric membranes can be designed to facilitate the passage of oil, but the more successful approach is with hydrophilic types that encourage the passage of water. Ceramic membranes have an advantage here as they are less often irreversibly fouled and give a higher recovery of oil, with a lower flux decline. Furthermore, they can be cleaned up by a simple heating procedure. More attention should be given to understanding the mechanism of fouling so that operating conditions can be optimised to further reduce fouling and further decrease the flux decline, as well as assisting in the design of antifouling membranes. Another obstacle to ceramic membrane use is the high cost of manufacture. Cheaper starting materials such as clays have been surveyed.

## 1. Introduction

With the growth of industry, the amount of oily wastewater is continuously increasing. Oily wastewater significantly impacts the environment by polluting drinking, groundwater and seawater resources [1,2,3,4,5,6]. As a result, the damage can be expanded to the whole ecosystem. To remove oil from water, many methods tested so far include, as well as membranes, electrochemical, biological, UV irradiation, hybrid technologies and destabilisation of emulsions by the addition of minerals. The development of a range of membranes has been highlighted, including those made by interfacial polymerisation, nanoparticles incorporation and surface grafting [7,8]. 

The feasibility of the membrane technology in oil-water separation has been confirmed by the application of varied types of membranes on different areas. For example, it was reported that the membrane separation process can be applied in the oil refining industry for deacidification and degumming [9]. Moreover, previous researches also reported the application of membrane technology to treat the oily wastewater from a vegetable oil factory [10], petroleum wastewater [11], and wastewater containing oil from other industries [12]. 

**Figure 1:**
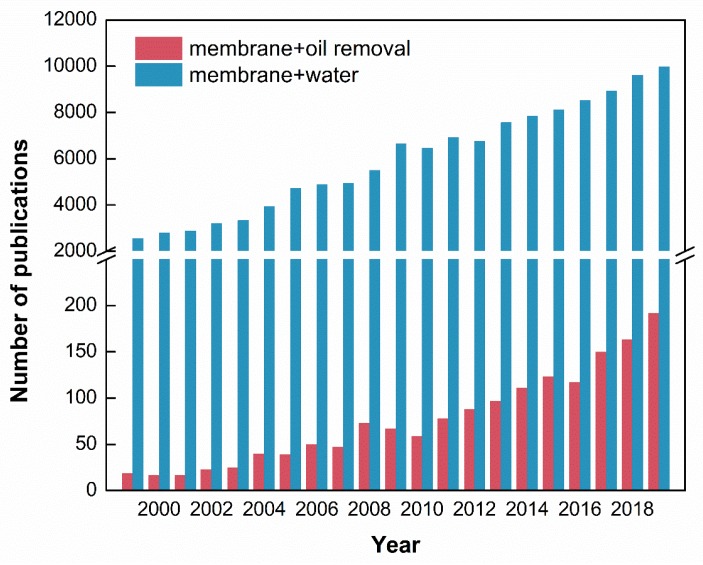
The total number of publications associated with membranes in the literature during 1999 to 2019.

According to the Scopus database, Figure 1 illustrates the number of annual publications from 1999 to 2019. When using “membrane” and “water” as the keywords, the number of publications increased from 2558 in 1999 to 9976 in 2019. The membrane technology is getting much attention with regards to oil removal from wastewater due to its advantages such as high-effective oil droplets removal, low energy consumption and mid required temperature. It could be found that, in 1999, only 19 publications were about oil removal of membranes. In 2019, the number of annual publications increased 10 times to 192. 

**Figure 2:**
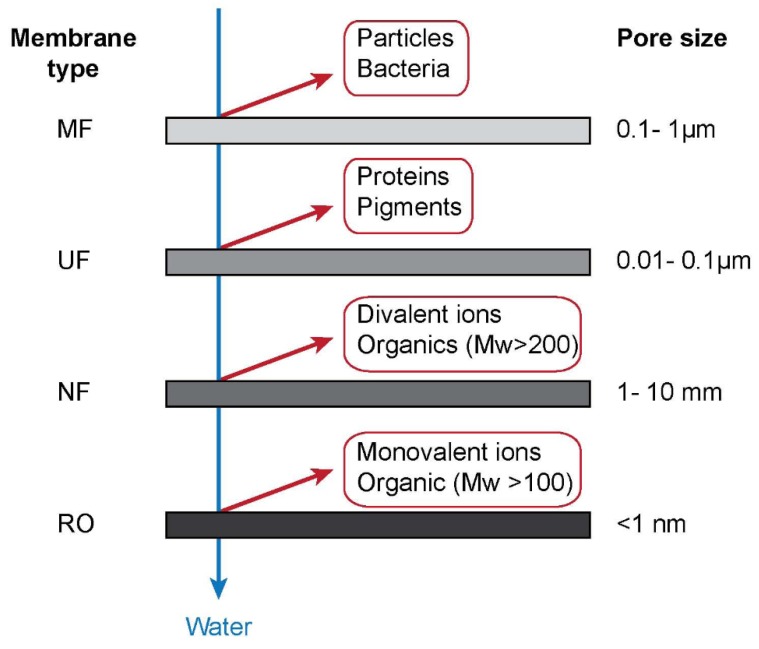
The classification of membranes, adapted from [19].

According to the pore size, membranes can be classified into microfiltration (MF), ultrafiltration (UF), nanofiltration (NF), and reverse osmosis (RO), as shown in Figure 2. Among these membranes, MF and UF are the major filtration processes used for oil removal. A wide-ranging review of advanced polymer membranes discusses diverse structures and functions of future membranes [13]. Among the trends predicted is creating tailored barrier or surface structures on membranes. These are relevant for effects produced by the chemical structure of the membranes which are the focus of the present review. Hydrophilic surfaces will repulse oil droplets from the membrane and facilitate the passage of water. The introduction of fluorine-containing species is another approach that is under study [14,15,16]. Ceramic membranes are also a very attractive system for the passage of water resulting in highly efficient oil removal [17,18].

Although the feasibility of the membrane technology was realized by researchers, the number of publications about oil removal by membranes accounts for a small percentage of the total publications associated with membrane technology. This mini review will provide a short summary on state-of-art advances in the membrane technology for oil removal from wastewater. 

## 2. Organic Membranes

**Figure 3:**
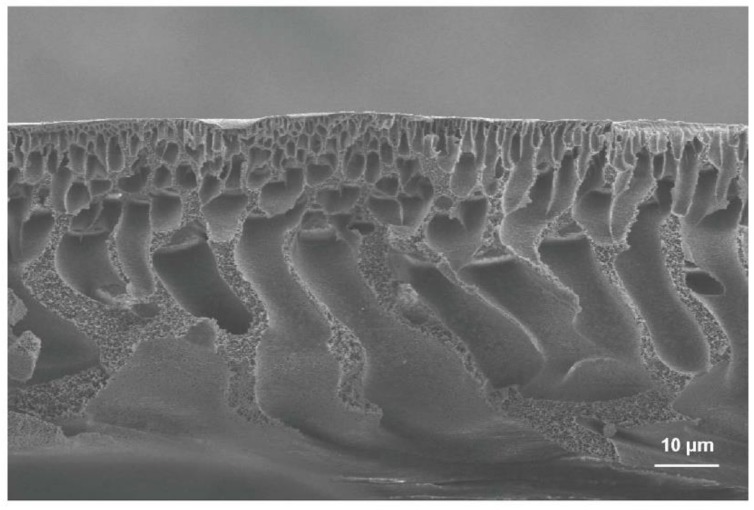
The typical cross-sectional SEM image of the PS membrane fabricated by the phase inversion process.

Polymers such as phlysophone (PS), polyethersulphone (PES), polyvinylidene fluoride (PVDF), polyacrylonitrile (PAN) and cellulose acetate (CA) were widely used to prepare MF and UF membranes. These membranes are usually fabricated by phase inversion. Figure 3 shows a typical cross-sectional SEM image of the PS membrane. The structure of the membrane contains a dense skin layer on the top, finger-like pores on the middle and sponge-like pores on the bottom.

As shown in Table 1, these membranes all have good performance for oil removal. During the oil removal process, the organic membranes shows advantages such as low-cost fabrication, easy processability, and low energy requirements. However, these membranes also exhibit drawbacks including relatively high tendency of membrane fouling, and a short lifetime. Therefore, researchers developed some approaches to further enhance the performance and properties of these membranes, including the optimization of running parameters and the improvement of membrane hydrophilicity.

### 2.1. Polysulphone

Fundamental studies of the ultrafiltration (UF) of stable oil-in-water emulsion by a series of commercial polysulphone (PS) UF membranes have been carried out [20]. Cellulose and polyamide UF membranes were also included in the studies. Experiments on droplet size and properties, flux and oil rejection by the membranes were performed. The molecular weight (MW) cut off varied from 5 to 30k for the cellulose membrane, 30k for the PS and 100k for the polyamide. Droplet size varied with concentration, oil to surfactant ratio, shear and interaction with the membrane. There was coalescence within the membranes, and a greater amount in the permeate. Oil rejection for all membranes for feeds containing at least 1000 mg/L of oil was high at >99.9%, with less than 20 mg/L remaining in the product water. The cellulose membrane was the easiest to clean and the PS membrane the most difficult.

Another early study compared results using PS, polypropylene and regenerated cellulose membranes with pore sizes of 0.02–0.2 µm [31]. The hydrophobic membranes allowed the passage of the oil phase and retained the water phase, even though the size of the water molecule was much smaller than that of the oil molecule. This has been ascribed to the blocking out of the water molecules by adsorption of non-polar molecules in the membrane pores [32].

Properties of PS membranes such as the porosity, pore size distribution and morphology of the membrane played a major role in determining the flux [21]. Membrane modification is an effective approach to adjust these properties. For example, it was reported that PS membranes have been given greater hydrophilicity and porosity by additives such as high MW polyvinylpyrrolidone and poly(ethylene glycol), resulting in oil retentions of >90% and residual oil levels of <10 mg/L [21]. The modified membranes were reasonably resistant to fouling, but a small amount of irreversible fouling was encountered. 

The operating parameters such as the temperature and pH of the feed solution, the pressure and flow velocities play a critical role in determining the filtration performance of membranes, as did alterations to the membrane properties. Mohammadi et al. reported a study about using commercial PS UF membranes with a 30 kDa MW cutoff to treat wastewater from a vegetable oil factory [10]. It was advocated that a high-pressure difference (>3 bar), high crossflow velocities, temperature of 30 °C and a pH level of nine was the optimized operating condition. The total organic carbon level was lowered from 300 to 39 mg/L. There was also an 85% reduction in the phosphate level. 

A polysulphone NF membrane has been used for cleaning up fuel oil wastewater, under varying conditions of oil content (10–40 mg/L) and temperature (15–40 °C), to determine the optimum conditions [22]. The best results were obtained when the oil content was 7 mg/L and the temperature was 31 °C, to give 100% removal at a flux of 65 L/m^2^h.

### 2.2. Polyethersulphone

Polyethersulfone (PES) membranes have been modified with the amphiphilic copolymer Pluronic F127, an amphiphilic poly(ethylene oxide)-poly(propylene oxide)-poly(ethylene oxide) block copolymer, for the purpose of separating oil/water emulsions [23]. Due to the enhanced surface hydrophilicity, a flux of 83 L/m^2^h was achieved. Oil droplets deposited on the surface of the membrane, causing serious membrane fouling, with no flux recovery on washing with water. However, there was a 93% recovery when the membranes were washed with sodium dodecyl sulphate solution. This was ascribed to the prevention of the coalescing of oil droplets on the membrane surface, thus allowing reuse of the membranes. 

The surface of PES membranes made by using polyvinylpyrrolidone (PVP) as a pore forming agent was been modified by corona air plasma and the membranes tested for oil removal [24]. The authors review the plasma treatment that UF membranes have been subjected to over the years to enhance surface hydrophilicity and permeation. There was an improvement in the oil/water flux, up to 70 L/m^2^h and in antifouling properties. The oil rejection was 98.2%–99.5%.

Blended PES/cellulose acetate/polyethylene glycol asymmetric membranes have been shown to have high permeability [26]. They had a thinner outer skin layer, higher surface porosity and larger pore sizes, with a mean pore size of 0.15 µm. There was stable oil rejection of 88% and a water flux of 27 L/m^2^h, versus ~7 L/m^2^h for the parent PES membrane.

PES UF membranes have been made in hollow fibre form by a phase inversion method [25]. PVP was added to the spinning dope in the ratio of 2.5 wt % to 17 wt % of PES. In separating oil from oily wastewaters of concentration 78 mg/L, an oil and grease removal of 99.7% was possible, at a flux of 84.1 L/m^2^h.

### 2.3. Poly(vinylidene difluoride)

In early work, the focus was on hydrophobic membranes such as poly(vinylidene difluoride) or PVDF. The permeation of oil through a PVDF membrane has been investigated [27]. Some 77% of the oil from an oil in water mixture containing 1% kerosene passed through the membrane. A related super hydrophobic membrane has been made from a fluorine-containing triptycene-based polyimide, which had a separation efficiency of oil from water of 99%, again with the oil phase passing through the membrane [28].

Poly(ethylene glycol) diacrylate (PEGDA) has been grafted onto the surface of a PDVF membrane via plasma induced graft polymerization [33]. The modified membrane was tested on oil in water emulsions and gave good separation efficiency and high fluxes at ultralow pressure, a range of oils giving water recovery efficiencies of 97%–99% and fluxes of 2420–3770 L/m^2^h. The membranes had excellent mechanical properties. 

Hydrophilic-oleophobic coatings have been explored as a way of producing self-cleaning surfaces [34,35]. There has been a search for surfaces that can simultaneously display hydrophilicity and oleophobicity such that the surface is strongly repellant to oil. This has focused on perfluorinated species in combination with a suitable hydrophilic polymer, as obtained by mixing aqueous solutions of poly(diallyldimethylammonium chloride) and sodium perfluorooctanoate [36]. This overcomes the fouling of the hydrophilic surface by oil.

A further example is the preparation of hollow fibre membranes by blending PVDF with a triblock copolymer AP that had both hydrophilic and oleophobic surface properties [14,15,16]. The triblock copolymer was made from vinylidene fluoride and chlorotrifluoroethylene [Poly(VDF-co-CTFE)] that was grafted with t-butyl methacrylate and the methacrylate groups then hydrolysed to form poly(methacrylic acid) units. The acid groups were subsequently esterified with a perfluoroalkyl-polyethylene glycol surfactant [fPEG to form P(VDF-co-CTFE)-g-PMAA-g-fPEG], denoted as AP. Depending on the level of content of AP, the resulting membranes had lower flux decays in treating oil/water mixes, ranging from 33% to 52%, and much higher flux recovery rates of 60% to 100%, all with a >99% removal of oil. The fouling could be easily removed by simple physical cleaning. Membranes where a modified AP that had no perfluorinated PEG and were highly hydrophilic were not effective, as was the parent PVDF membrane, which became seriously fouled.

Polyvinylpyrrolidone has been grafted onto a PVDF UF membrane to produce a membrane suitable for oil removal [37]. The average pore size increased slightly, and the performance in oil removal increased significantly. Fouling was the main cause of flux decline, but it could be 90% reversed by cleaning with 3 wt % caustic soda. Many other methods of improving hydrophilicity have been explored. An example is the introduction of hydrophilic cellulose nanocrystals which increase the pure water flux some 20 times [38].

The modification of PVDF membranes by introducing hydrophilic structures to both encourage water passage and minimise fouling has been comprehensively reviewed [39]. More recently, surface hydrophilic modification has been achieved by using tannin or polyethyleneimine [40].

### 2.4. Polyacrylonitrile and Polyamide

The operating conditions for commercial polyacrylonitrile UF membrane with a highly hydrophilic structure has been optimised for use at 45 °C [41]. There was a 100% removal of oil and grease initially, but there was a flux decline caused by fouling via both pore blocking and cake layer formation. Fouling resistance was improved by operating at a higher than usual temperature.

An electrospun nanofibrous membrane has been made by depositing polydopamine nanoclusters onto a crosslinked polyacrylonitrile/hyperbranched polyethyleneimine (PDA/PAN/HPEI) membrane [8]. The membrane exhibited high permeate flux for the removal of oil from oil/water emulsions separation and had excellent recyclability. It was able to separate ~98.5% of the oil with a high flux of about 1600 L/m^2^h.

A commercial polyamide thin film composite UF membrane has been tested under varying conditions of oil content (10–40 mg/L) and temperature (15–40 °C) for the optimum purification of fuel oil wastewater [22]. Oil removal was >96% at a flux of 624 L/m^2^h, which was strongly influenced by the applied pressure.

### 2.5. Cellulose

Compared to PS-based membranes, Cellulose membranes shows typical advantages such as easy-cleaning [20]. A hydrophilic hollow fibre UF membrane composed of cellulose has been fabricated using as solvent a mixture of N-methylmorpholine-N-oxide and polyethylene glycol [29]. In treating 800 mg/L of machine oil in water the oil retention was over 99% and the residue was <10 mg/L. The membrane was resistant to fouling and tolerant over a wide pH range, from 1–14.

A regenerated cellulose UF membrane has been modified by grafting poly(N-isopropylacrylamide) block poly(oligoethylene glycol methacrylate) nanolayers onto the membrane surface [42]. This led to a ~40% decrease in the water flux, but this was comparable to commercial membranes used for the removal of organic compounds. This indicated that the modified membranes could be used to separate large volumes of oils from emulsions at high flux. The removal of organics was >97%.

Membranes fabricated based on cellulose acetate (CA) often show high hydrophilicity, high water permeability and reduced membrane fouling tendency. The feasibility of CA-based membranes in oil removal from wastewater has been confirmed by a research from Nanyang Technological University (NTU), which indicated that the CA hollow fibre UF membranes exhibited high performance, low energy consumption and minimized membrane fouling [30]. Another research reported a hydrophilic cellulose acetate UF membrane applied to treat oilfield wastewater [38]. A 98.3% removal of oil and grease was achieved from a raw water containing 230 mg/L of oil and grease. A novel membrane has been reported by grafting polyacrylonitrile (PAN) on to CA membranes [43]. This modification changed the morphology of membranes and improved the performance of CA membranes. 

## 3. Inorganic Membranes

An extensive review of inorganic membranes covering their preparation and use in water treatment generally has been published recently [44]. The point is made that most membranes in practical use are based on organic polymers. These have their deficiencies because of their low mechanical strength, lack of thermal stability and fouling tendency. Membranes fabricated by inorganic materials such as alumina, zirconia, titania, silica, silicon carbide and clay mixture have been reported for removing oil from water (Table 2). Inorganic membranes have good mechanical properties and long-term chemical and thermal stability. Hence regeneration can be achieved by heating. They also have a relatively narrow pore size distribution and higher porosity, resulting in better separation characteristics and a higher flux, leading to a lower membrane area needed and a smaller footprint. However, the high fabricating cost is one of the biggest challenges for the large-scale application of inorganic membranes.

Early work on a comparison with organic polymers made use of hydrophilic PVDF and hydrophilic PS, which had pore sizes of 0.45 and 0.1 μm respectively. Both had flat sheet formats. The inorganic membranes were hydrophilic commercial products: Ceramesh, a flat plate zircon-coated nickel alloy mesh, and Membralox IT-70, which had a tubular format [59]. Both had a 0.1 μm cut off. In treating a 1000 mg/L emulsion, these MF membranes the ceramic membranes gave superior performance, giving a higher and more stable flux. They were also less susceptible to fouling and easier to control. Since then, the application of inorganic membranes in the treatment of oily water, usually in MF and UF applications, has been tested with several different membranes. 

### 3.1. Alumina

Two commercial α-Al_2_O_3_ membranes have been tested for oil removal. They had pore sizes of 0.2 and 0.8 μm in a surface layer that was 4–5 μm thick [45]. About 98%–99% oil removal was achieved. Significant fouling and flux decline were observed. For a 250 mg/L feed the final flux was 30–40 L/m^2^h.

A layer of α-Al_2_O_3_ has been deposited on the outer surface of a cordierite supporting tube by dipping, cordierite being a magnesium/iron/aluminium cyclosilicate [18]. A similar membrane was made using γ-Al_2_O_3_. The aim was to produce high flux membranes. The final product had a pore size of ~100 nm and a porosity of ~70%. In the treatment of a synthetic bilge water containing 4000 mg/L of oil it was possible to obtain a final oil content of only 15 mg/L. Any oil deposited on the membrane could be easily removed by heating to 500 °C for 2 h, when the membranes were regenerated without any structural change.

A similar approach has been made with a tubular α-Al_2_O_3_ membrane [46]. An oily wastewater of up to 2000 mg/L was tested. Removals of 96%–98% were possible, with the flux reaching a peak of 163 L/m^2^h at pH 5.8. The salt concentration also had an influence, with a higher concentration of 0.05 mol/L giving a flux of only 45% of that for a lower concentration of 0.001 mol/L. However, the oil removals were little different, at 97.2% and 97.7%. The trans-membrane pressure was a significant variable. When it was increased from 0.05 to 0.30 MPa, the flux improved from 30 to 210 L/m^2^h, but there was greater fouling. For some wastes the membranes have been found to be prone to fouling by waxes and asphaltenes [17]. Further good oil removal results have been obtained using an alumina membrane, with 95%–99% removals of oil [47,48]. A commercial tubular α-Al2O3 MF membrane with a pore size of 0.2 μm has been used to remove 95% of the oil from a refinery effluent, operated at 32.5 °C, leaving but 4 mg/L in the permeate [49]. Backwashing prevented a significant decline in flux caused by the presence of oil droplets and particulates blocking the membrane pores.

A membrane comprised of a thin mesoporous γ-Al_2_O_3_ layer 500–1000 nm thick on a macroporous α-Al_2_O_3_ support has been prepared, and a study made of pore size and surface chemistry effects [60]. An analogous system with silica particles of the MCM-48 type was also made which had a thickness of ~30 nm. The permeability of the hydrophobic hexane and toluene through the γ-alumina composite membrane was lower than that of hydrophilic alcohols and water. The silica version had a higher permeability than γ-alumina, which was attributed to its much smaller thickness.

A γ-Al_2_O_3_ multilayer UF membrane has been prepared on an α-Al_2_O_3_ supporting membrane by a sol-gel process [50]. In the treatment of a real wastewater from a refinery, 79% of the oil and grease could be removed at a flux of 113 L/m2h when the operating temperature was 35 °C.

### 3.2. Zirconia

MF membranes based on ZrO_2_ and composed of a 30 μm layer with a pore size of 0.2 μm have, after conventional flocculation, resulted in a product containing 8.8–10.8 mg/L of oil from a wastewater of 6000 mg/L initial concentration [51]. The flux ranged from 120 to 170 L/m^2^h depending on whether there was a prior flocculation step.

ZrO_2_ UF membranes have been coated with the metal oxides TiO_2_, Fe_2_O_3_, MnO_2_, CuO, and CeO_2_, which had a size of around 10 nm, using a pulsed laser deposition procedure. The resulting membranes were tested for oil recovery from aqueous emulsions [61]. It was found that the higher the hydrophilicity of the deposited oxide, the lower the irreversible fouling tendency of ceramic membrane, in the order Fe_2_O_3_ < TiO_2_ < CuO < CeO_2_ < MnO_2_. Hence the hydrophilicity of the deposited metal oxide was a major factor in lowering the fouling of the zirconia membrane.

An alumina MF membrane has been coated with highly hydrophilic zirconia nanoparticles using ZrCl_4_ as the precursor [62], as quoted by [15]. There was an improvement in flux when treating oil in water emulsions, relative to the parent membrane.

A crossflow ceramic MF system based on a ZrO_2_–TiO_2_ membrane of 0.45 μm pore size has been explored for cleaning up surfactant-stabilised emulsions from oil exploration, alone or in combination with 1.5 g/L of colloidal bentonite [52]. The latter addition caused a severe decrease in permeability that was 3.5–5 times that for the emulsion alone. The initial wastewater contained 10,000–24,000 mg/L of total organic carbon, which was reduced by 79%–91%. Membrane cleaning was essential after every run and was based on a published procedure involving a water wash followed by alkali treatment at 80 °C [63].

### 3.3. Titania

Early work on oilfield wastewater with membranes made use of tubular alumina systems coated with TiO_2_ particles on the internal surface to produce ceramic MF, UF and nanofiltration composite membranes to produce a final cut off MW of 750 Da [53]. Oil removal was up to 99.5% for feeds ranging from 32 to 5400 mg/L.

A tubular porous carbon support membrane when coated with TiO_2_ particles of size ~1.7 μm removed 98% of the oil from a 333 mg/L oil in water emulsion, giving a 98% rejection and a product water containing only 8.3 mg/L of oil [54]. On heating from 20 to 70 °C, the flux increased 85 times to over 210 L/m^2^h.

A nano-TiO_2_ coating has been inserted into a commercial tubular alumina MF membrane and the combination tested on a waste oil in water emulsion [64]. The hydrophilic coating prevented oil droplets from penetrating the membrane pores, so the modified membrane had a higher flux than the unmodified membrane. The effect of the nano-TiO_2_ coating on the membrane surface is weakened if an oil cake layer is formed when the feed has a high oil concentration. It was found that oil droplets penetrated the membrane pores and covered the oxide particles during the separation process.

Two commercial TiO_2_ membranes, MF and UF types, have been studied for oil removal, along with other inorganic membranes [63]. The initial fluxes were 150–200 and 50–100 L/m^2^h, respectively. There was a need for improved membrane cleaning if the flux was to be maintained.

In order to reduce membrane cost, coal fly ash was effectively recycled for the first time to fabricate mullite hollow fibre with finger-like and sponge-like structures, on which a much more hydrophilic TiO_2_ layer was deposited [55]. Mullite is a rare silicate mineral that is produced during various firing processes; it has two stoichiometric forms: 3Al_2_O_3_.2SiO_2_ or 2Al_2_O_3_.SiO_2_ and can be used as a refractory material. A composite ceramic hollow fibre microfiltration membrane made in this way was tested for oil removal and found to remove 92%–97% of the organic carbon from an emulsion containing 200 mg/L of oil. The membrane was easily cleaned with dilute alkali to effectively accomplish membrane regeneration with a ~98% flux recovery.

MF membranes have been made from fly ash and TiO_2_, followed by sintering at 1100 °C. With an increase in titania content, the pore size was reduced from 3.0 to 1.3 nm [65]. There was a maximum oil rejection of 99%, obtained with the membrane of smallest pore size.

### 3.4. Silica

A silica MCM-48 membrane has been made on an α-alumina porous support using a hydrothermal technique [66]. Silylation with trimethylsilane and triethylsilane was used to enhance the stability and hydrophobicity of the membrane. Silica MCM-48 has a three-dimensional pore structure which is very regular, and a pore size distribution that is nearly as sharp as that of conventional zeolites [67]. The membranes were used in studies of the separation of ethanol/water mixtures. They could be candidates for oil removal systems, warranting further study.

### 3.5. Silicon Carbide

Commercial MF and UF membranes based on silicon carbide of pore sizes 0.5 and 0.04 μm were used on wastewater samples taken from oil platforms operating in the Arabian Gulf. [63]. The initial fluxes were high at 2000–2700 and 630–780 L/m^2^h, respectively, at a flow rate of 1.6 m^3^/h. The membranes outperformed analogous TiO_2_ membranes despite their higher porosity of up to 2 μm, when operated under the same conditions. However, the SiC membranes also had a higher tendency to foul because of their more open pores, so they required effective chemical cleaning. Oil rejections on feeds containing 5–45 mg/L were ~70%.

### 3.6. Clay Mixtures

A tubular MF membrane made from kaolin by calcining it at 900 °C had a pore size of 10 μm. It was not as susceptible to fouling as organic membranes when treating oil—water emulsions of a concentration less than 2000 mg/L as it was very hydrophilic in nature [68]. The flux increased with increasing temperature and pressure. With increasing oil content, the membrane rapidly fouled, but not excessively. There was an initial flux reduction because an oily layer formed on the membrane surface, but its thickness did not increase with time because of the action of the flow. Operation at temperatures of less than 40 °C was recommended as higher temperatures increased the processing cost.

Inexpensive inorganic precursors such as kaolin, quartz, calcium carbonate, sodium carbonate, boric acid, and sodium metasilicate have been employed in the preparation of an MF membrane for the treatment of oily wastes [56]. The average pore size was 0.55 μm. In treating a 50 mg/L emulsion a 97% rejection of oil was exhibited. Pore blocking caused a decrease in flux, followed by cake filtration. Similarly, mullite and mullite-alumina MF membranes have been made using kaolin [58]. That with 50% alumina was the best performer, with a flux of 105 versus 73 L/m^2^h for the 100% mullite one on treating a synthetic wastewater. These fluxes were reduced to 58 and 41 L/m^2^h for a real wastewater. Recoveries were 94% and 84% over arrange of wastewater levels of 250–3000 mg/L. The rejection of total organic carbon by these membranes could be as high as 94%. The membranes have been used in studies of the fouling mechanism [69].

Low cost MF membranes have been made by sintering various mixtures of clay, quartz, calcium carbonate and titania [57]. They had pore sizes of 0.45–1.30 μm. The best was a 50/25/22/3 mix which had a good combination of flux and rejection, giving an 87% oil rejection when treating an oil in water emulsion of 100 mg/L. A later study showed that as the TiO_2_ content increased the porosity and mechanical stability of the membranes improved and the average pore size was reduced [65]. Such membranes gave a 99.2% recovery from a 200 mg/L wastewater.

## 4. Hybrid Inorganic—Organic Membranes

The topic of polymeric membranes impregnated with a variety of nanoparticles has been reviewed [70]. It covers the broad use of membranes containing silver, iron, zirconium, silica, aluminium, titanium and magnesium-based nanoparticles. Oil removal is not specifically mentioned. Table 3 listed some typical hybrid inorganic—organic membranes, it was noted that polymeric membranes modified by adding such nanoparticles can have increased permeability, less fouling, a higher tensile strength, a higher selectivity for certain compounds, a better performance over a wider temperature and pH range, and a higher diffusion rate. Silver and titanium-based nanoparticles can reduce biofouling. However, in drinking water applications, care needs to be taken regarding the toxicity of the nanoparticles; silica would be the most benign of them.

### 4.1. TiO_2_/Poly(vinylidene difluoride)

Modifying PVDF membranes by attachment of hydrophilic species to the membrane surface so that the oil is inhibited in its passage, allowing only water to pass through, has been applied to inorganic as well as organic species. A study has confirmed that TiO_2_ nanoparticles can be installed on the surface of a PVDF membrane with LiCl.H_2_O also present, making the surface of that hydrophobic polymer membrane quite hydrophilic [73]. Much better flux and oil rejection was then achieved, at 82.5 L/m^2^h and 98.8%, respectively.

Hollow fibre PVDF membranes containing 2 wt % of TiO_2_ and 5 wt % of polyvinylpyrrolidone (PVP) have been prepared for treating oily wastewater [74,75]. When treating 250 mg/L oil water the best results were with PVP of MW 40kDa, when the oil rejection was 94% and the flux peaked at 72 L/m^2^h. A simple back flushing resulted in a 60% recovery of the flux. The composite PVDF membrane showed better performance compared to a PVDF membrane without TiO_2_.

With the incorporation of Al_2_O_3_ as well as TiO_2_ and with the presence of an anionic polyacrylamide as well, similar results were obtained, namely residual oil levels of <50 mg/L being obtained at a flux rate of 70–160 L/m^2^h [77,78].

In other work a silane coupling agent has been used to bind the nanoparticles to the membrane surface [76]. The resulting membrane was tested for the separation of oil from oil in water emulsions and found to give a nearly 99% recovery, with fluxes in the range of ~350–600 L/m^2^h. It was reported to have good oil resistance and antifouling properties. 

A superhydrophilic UF membrane has been prepared by incorporating TiO_2_ nanoparticles throughout the membrane [82]. It was tested for humic acid rejection and flux decline, giving good performance on both, as well as a high rejection. Its life could be extended by a simple physical cleaning.

### 4.2. Al_2_O_3_/Poly(vinylidene difluoride)

A tubular PVDF UF module that had been modified with nano-sized alumina particles has been applied to the removal of oil from a dilute wastewater that had a total organic carbon content of 15.5 mg/L [72]. The flux was 160 L/m^2^h, versus ~80 for the unmodified membrane. The antifouling properties were also improved. There was essentially a full recovery of flux after washing the membrane with a 1% surfactant solution at pH 10.

### 4.3. SiO_2_/Poly(vinylidene difluoride)

Another approach to making what the authors describe as superhydrophobic and superoleophilic PVDF MF membranes makes use of silica nanoparticles modified with hexamethyldisilazane [(CH_3_)_3_Si]_2_NH via a dip coating method [79]. In oil and water mixture separation the membranes had a relatively high flux of ~2500 L/m^2^h, higher than the 300 L/m^2^h obtained with commercial PVDF membranes. The high flux was maintained after 10 cycles, and if after that cleaning was required, a simple ethanol wash recovered the flux. To test whether electrostatic adsorption or chemical bonding of a fluorinated silica to PVDF gave the better anti-fouling performance, a comparison of the two methods was made [80]. The fluoridated silica was 1H,1H,2H,2H-perfluorodecyltriethoxy-silane. In direct contact membrane distillation tests of coking wastewater, the fluoridated version was preferred. It gave excellent anti-fouling performance and had less flux decline than the parent PVDF membrane.

### 4.4. SiO_2_/Polyethersulphone

Mixed matrix membranes have been made by filling PES membranes with hydrous manganese dioxide nanoparticles [71]. Due to the presence of hydroxy groups, membrane hydrophilicity and the resistance to fouling by oil were improved in treating oil/water mixtures. The water flux was 100–150 L/m^2^h, versus 39 L/m^2^h for PES itself. Flux recoveries were 93% for a 100 mg/L feed and 75% for a 1000 mg/L feed.

### 4.5. Clay/Cellulose Acetate

Clay has been used to create a composite membrane with cellulose acetate [81]. Obtained from the River Ganges, the clay comprised the ceramic support, onto which cellulose acetate was cast by dip coating. UF and MF style membranes were prepared that had pore sizes of 0.56 and 0.28 nm. On treating oil in water emulsions containing up to 200 mg/L of oil, a 93% removal was possible. Because of the low cost of the ingredients, the total cost of the membrane was much less than that of commercial membranes.

### 4.6. Carbon-Based Nanomaterials/Polymer

Carbon-based nanomaterials such as activated carbon, graphene oxide (GO), and carbon nanotubes (CNTs) have been used to modified polymer membranes. Gu et al. fabricated a hybrid CNTs/polystyrene membrane. The membrane exhibits a high water flux as 5000 L/m^2^hbar, and high separation efficiency [83]. In another research, a hybrid GO/aminated polyacrylonitrile membrane was fabricated by modifying the surface of the aminated polyacrylonitrile electrospun membranes with GO nanosheets. The membrane shows improvement in hydrophilicity, porosity which enhanced the water flux to as high as 10,000 L/m^2^h. Moreover, the connection between GO nanosheets and the polymer also improved the oil rejection of the membranes [84].

### 4.7. Metal-Organic Frameworks (MOFs)/Poly(lactic acid)

MOFs are coordination polymers comprised of a ligand having at least two coordinating groups to form a bidentate structure. They are ligand-metal alternating copolymers and can exist as a three-dimensional format with crosslinking through the metal. There is a wide range of choices in the balance of organophilic and hydrophilic structures within the membranes, and also in the search for membranes with very large pores. Membranes of this type have been comprehensively reviewed [85,86], with the main application being gas separation [87].

An organophilic membrane has been made from zinc(2-methylimidazolate)_2_, which is known as ZIF-8. ZIF-8 is chemically and thermally stable, and remarkably resistant to water and organic solvents [88].

MOF-based mixed matrix membranes for liquid separations have been reviewed [89]. In the one study on oil/water separation that is reported, the nanoparticles of ZIF-8 have been incorporated into poly(lactic acid) or PLA to prepare an electrospun nanocomposite membrane [90]. The PLA/ZIF-8 membrane showed increased oil wettability, significantly better mechanical properties and a much higher separation efficiency than the parent PLA membrane, indicating that it could be a good candidate for oil/water separation. The oil wettability and mechanical properties of PLA itself are not ideal, which limits its wide application [91]. There is clearly a need for more research on MOF-based mixed matrix membranes for oil recovery.

## 5. Comparison of Various Systems

The results obtained with the organic, inorganic and hybrid organic/inorganic membranes are summarised in the Tables. Direct comparisons of the three types of membranes have been made for the same wastewater. In one example, a ceramic α-Al_2_O_3_/ZrO_2_ and an organic polymer membrane fabricated from poly(vinyl chloride) or PVC were examined [92]. The latter was selected for its ready availability and low cost. The better result was with the ceramic one because of the reversibility of fouling and the higher oil removal efficiency.

Another comparison made use of four ceramic membranes—Al_2_O_3_, ZrO_2_, TiO_2_ and SiC—and a PES/PVP MF membrane [93]. Reversible fouling followed the order PES/PVP ~ Al_2_O_3_ ~ ZrO_2_ > TiO_2_ > SiC, while for irreversible fouling it was PES/PVP > ZrO_2_ > Al_2_O_3_ > TiO_2_ > SiC.

**Figure 4:**
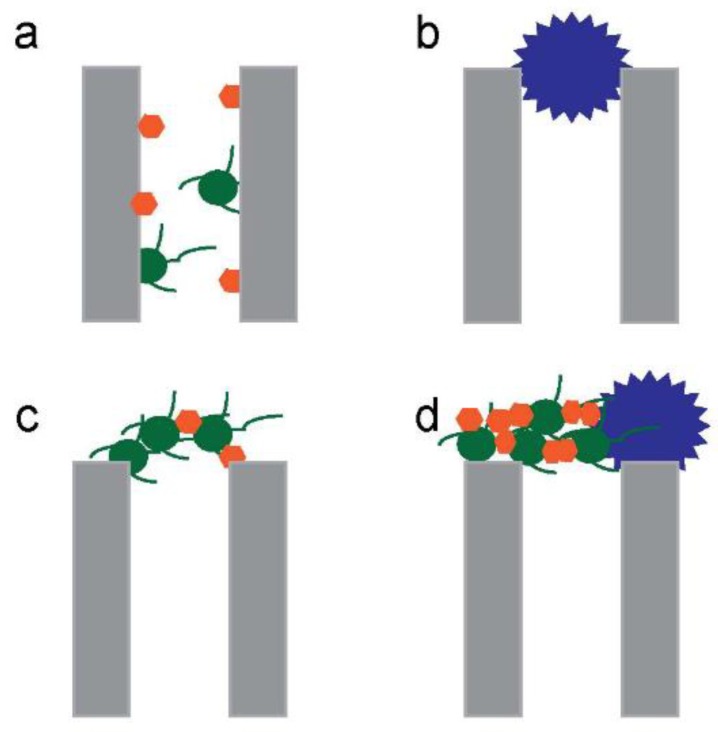
Different mechanisms of membrane fouling. (**a**) standard blocking, (**b**) complete blocking, (**c**) intermediate blocking and (**d**) cake layer formation.

Later discussions highlight that fouling is a major problem with the membrane processes. This requires the use of very stable membrane structures so that rigorous cleaning can be applied, which favours inorganic structures [94]. That fouling greatly reduces the effectiveness of membranes for oil removal has been highlighted in recent works [95]. Researchers modelled the fouling mechanisms of mullite MF membranes during different membrane stages, including the standard blocking, complete blocking, intermediate blocking, and cake layer formation (Figure 4). The result showed that the cake filtration model can well predict the flux decline [69]. Increasing the pressure from 0.5 to 4 bar decreased the porosity of the cake layer from 25.7% to 15.0%. After the cake filtration model, an intermediate pore blocking model was found to well predict the results. The cake/gel layer was also found to be the best model for the optimum purification of fuel oil by a polyamide thin film composite UF membrane, whereas with a PS NF membrane the fouling mechanism followed the cake filtration model [22].

A review of the colloidal aspects of membranes for oil removal includes a discussion of fouling mechanisms [96]. The problem is made more difficult with oily feeds because of the complex range and number of compounds that are present. As a result, up to four different processes, including pore and surface blocking, and cake filtration, can take place at the same time. Although many anti-fouling membrane structures have been proposed, it has been emphasized that the top priority should first be for a better understanding of the mechanisms involved [3]. A study of wastewaters that are difficult to treat, using PS and cellulose UF membranes, has focused on the mechanism and control of fouling [97]. The early stage of fouling was by cake formation, which led the way to cake layer growth. The layer was highly compressible. A higher applied pressure is needed, but this does not necessarily mean higher flux results, as it can cause rapid fouling and a higher need for increased applied pressure. The threshold flux was around 15 L/m^2^h. Operating just below this was found to maximize yield while keeping fouling rates at acceptable levels. The study provided valuable insights into establishing best practices for cleaning-in-place systems.

The requirement for lower cost membranes has been stressed. The advantages and disadvantages of ceramic membranes more generally have been summarised in Table 4 [98]:

## 6. Conclusions

The removal of oil from wastewater is critical due to the severe damage of oily wastewater to the environment. Membrane technology has attracted increasing attention in this field. A considerable amount of work has been published on testing membranes for oil removal from wastewaters. From the few early efforts with hydrophobic organic membranes which allowed the passage of oil, to the preferred approach of hydrophilic membranes of all descriptions, organic, inorganic and hybrids of the two, where the water passes through the membrane. Organic membranes have advantages such as low-cost for fabrication, easy processability, and low energy requirements. However, these membranes also exhibit drawbacks including a relatively high tendency of membrane fouling, and a short lifetime. Many inorganic species have also been investigated, from metal oxides and carbides to metal-organic frameworks. Direct comparisons have shown that excellent results can be obtained with ceramic membranes because of the reversibility of fouling, and the higher oil removal efficiency. In addition, there is a lower flux decline. However, the manufacturing cost is an obstacle to their expanded use, so further ways of reducing it should be sought. It is essential that more work be carried out on the mechanism of fouling, to provide an improved basis for designing anti-fouling membranes.

## Figures and Tables

**Table 1:** Oil removal results using organic membranes.

Membrane Structure	Modifiers	Feed Oil Conce., mg/L	Oil Rejection, %	Residual Oil, mg/L	Water Flux, L/m^2^h	Reference
PS	Nil	1000	>99.9	<20		[20]
	Nil	300	87	<30		[10]
	PVP & PEG ^1^		>90	<10		[21]
		7	100		65 at 31°	[22]
PES	Pluronic F127				83	[23]
	PVP		98.2–99.5			[24]
	PVP	78	99.7	2	84.1	[25]
	Cellulose acetate/PEG		88		27	[26]
Polyacrylonitrile	PDA/HPEI ^2^		~98.5		1600	[8]
Polyamide		10–40	>96		624	[22]
PVDF	Nil	10,000	Permeate is oil 77	2300		[27]
	PEGDA ^3^		97–99		2420–3770	[28]
	Perfluorinated PEG triblock	Up to 115,000	>99	1–3	59–69	[16]
Flurorinated Triptycene			Permeate is oil ~99			[28]
Cellulose	Nil	800	>99	<10		[29]
Cellulose acetate	Nil	230	98.3	4		[30]

^1^ PVP is polyvinylpyrrolidone & PEG is poly(ethylene glycol); ^2^ PDA is polydopamine and HPEI is hyperbranched polyethyleneimine; ^3^ PEGDA is poly(ethylene glycol) diacrylate.

**Table 2:** Oil removal results using inorganic membranes.

Membrane Structure	Feed Oil Concn., mg/L	Oil Rejection, %	Residual Oil, mg/L	Water Flux, L/m^2^h	Reference
α-Al_2_O_3_	250	98–99	3–5	30–40	[45]
α-Al_2_O_3_	2000	96–98	40–80	163	[46,47,48]
α-Al_2_O_3_	75	95	4		[49]
γ-Al_2_O_3_	4000	99.6	15		[18]
γ-Al_2_O_3_ on α-Al_2_O_3_ support	Refinery waste			113	[50]
ZrO_2_	6000	99.8	9–11	120–170	[51]
ZrO_2_-TiO_2_	10,000–24,000	79–91			[52]
TiO_2_	32–5400	99.5			[53]
TiO_2_	333	98	8.3	85 at 20 °C, 210 at 70 °C	[54]
TiO_2_ on mullite support	200	92–97	6–16		[55]
Clay mixtures	50	97	1.5		[56]
Clay mixtures	100	87	13		[57]
α-Al_2_O_3_ and mullite	250–3000	84–94		58–105	[58]

**Table 3:** Oil Removal results using organic/inorganic hybrid membranes.

Membrane Structure	Modifiers	Feed Oil Concn., mg/L	Oil Rejection, %	Residual Oil, mg/L	Water Flux, L/m^2^h	Reference
PES	MnO_2_	1000	~100		Good recovery	[71]
PVDF	Al_2_O_3_	15.5	98	<1	160	[72]
	TiO_2_		98.8		82.5	[73]
	TiO_2_ and PVP ^1^	250	94	15	72	[74,75]
	Silane/TiO_2_		99		~350–600	[76]
	TiO_2_/Al_2_O_3_	200		<50	70–160	[77,78]
	SiO_2_				2500	[79]
	Fluorinated SiO_2_	Coking wastewater			18–20	[80]
Cellulose acetate	Clay	200	93	14		[81]

^1^ PVP is polyvinylpyrrolidone.

**Table 4:** Advantages and disadvantages of ceramic membranes.

Advantages	Disadvantages
Very high flux	High production cost
High mechanical, chemical and thermal stability	Low area to volume ratio
Long term stability under high temperatures	Brittleness
Lower tendency for fouling, easy cleaning after fouling	Low selectivity in large scale
Operate under high pressures, resist high pressure drops	Microporous membranes
Withstand harsh chemical environment, resist corrosion	Low permeability but high selectivity of dense membranes
No microbiological degradation	
